# Impediments to communication and relationships between infertility care providers and patients

**DOI:** 10.1186/s12905-018-0572-6

**Published:** 2018-06-05

**Authors:** Robert Klitzman

**Affiliations:** Columbia University, 1051 Riverside Drive #15, New York, NY 10032 USA

**Keywords:** Provider-patient relationships, Provider-patient communication, Infertility treatment, Fertilization, In vitro, Ethics, Policy, Education, Medical, Empathy, Patient-centered care

## Abstract

**Background:**

Infertility patients generally see provider-patient communication and relationships as important, but as often insufficient, raising critical questions regarding why these gaps persist, and how they might best be addressed.

**Methods:**

Semi-structured interviews of approximately one hour each were conducted with 37 ART providers and patients (17 physicians, 10 other health providers, and 10 patients) and were thematically analyzed.

**Results:**

Patients see clinicians’ interactions as ranging widely from good to bad, related to several specific barriers and factors. Patients and providers may differ in their physical and emotional experiences, expectations concerning treatment outcomes and uncertainties, and time frames and finances, generating dynamic processes and tensions. Characteristics of particular providers, clinics and patients can also vary. Infertility patients tend to find only one outcome acceptable – a “take home baby” – rather than partial success, as is the case with many other diseases. Yet most IVF cycles fail. Many patients must pay considerable out-of-pocket expenses for infertility treatment, exacerbating disappointments and frustrations. Providers often work in competitive, entrepreneurial markets, and “hype” their potential success. After treatment failures, providers may feel guilty and withdraw from patients. Yet these behaviors can antagonize patients more than physicians realize, aggravating patient stresses. Several providers described how they understood patients’ needs and perceptions more fully only after becoming infertility patients themselves. Interactions with not only physicians, but other providers (e.g., nurses and staff) can play key roles. Patients may be willing to understand these impediments, but providers often communicate these obstacles and reasons poorly or not at all, furthering tensions.

**Conclusions:**

These data, the first to examine several critical aspects of challenges that infertility providers and patients face in communication and relationships, suggest that several key dynamic processes and factors may be involved, and need to be addressed. While prior research has shown that infertility patients value, but often feel disappointed in relationships with clinicians, the present data highlight several specific impediments, and thus have critical implications for future practice, research, guidelines and education.

**Electronic supplementary material:**

The online version of this article (10.1186/s12905-018-0572-6) contains supplementary material, which is available to authorized users.

## Background

Infertility patients generally see provider-patient communication and relationships as critical, but often as insufficient, raising critical questions about why these gaps persist and how they might be best addressed. Many individuals are infertile, but face various obstacles in obtaining optimal infertility treatment; and examining these barriers is thus of importance.

Quantitative surveys have suggested that patients choose IVF clinics based on both published success rates and quality of service [1, 2]. In general, challenges in provider-patient relationships may result partly from the fact that physicians focus on evidence-based medicine, which is doctor-centered, with doctors interpreting scientific data, while patients are more concerned with their own individual needs, preferences and experiences [3]. Research has also examined the related concept of Patient-Centered Care (PCC) – i.e., that patients want to be treated as people, with respect for their values, preferences, needs and education. Studies have described how patient-centered infertility care has ten dimensions, related both to the system (information, competence, coordination, accessibility, continuity, and physical comfort) and human factors (staff attitude and relationships, communication, privacy and support) [4]. Dutch patients, for instance, often perceive weakness in their fertility care [5], including inadequate information regarding long term consequences of treatment (59%), lack of clarity about which interventions are reimbursed (50%) and about whom to contact for problems at nights and on weekends (54%), “no transparency in quality/performance” of clinics (61%), “too much time before a treatment plan was provided” (47%), and physicians not “deal[ing] well” with treatment-related anxiety and depression (40%).

Yet, clinicians underestimate the value to patients of patient-centeredness [6], while overestimating the value of ‘continuity’ of providers, and significantly misjudging several aspects of care, including the comprehensiveness of treatment information [7]. Physicians value patient-centeredness less than patients do. Patients would trade off 9.8% of pregnancy rate to see a friendly and interested doctor, instead of an unfriendly and uninterested one [5]. Researchers have concluded that fertility clinics should be more patient-centered.

Yet infertility providers can face various stresses related to the organizational aspects of the clinic [8, 9], contributing to patients feeling dissatisfied [2, 10]. Embryologists, for instance, engage in “emotional labor” regarding difficult patients, and giving “bad news” to patients [11]. In Europe, providers often work in either publically-funded infertility care (e.g., through the UK’s National Health Service), or in private clinics, and each setting may pose different sets of challenges (e.g., regarding patients on waiting lists at NHS clinics [12]). In the US, ART is not publically funded, and hence “public” clinics and this distinction (of publically-funded vs. private) do not exist. Yet US patients can consequently face considerable stresses paying for infertility treatment [13].

Major questions emerge regarding the implications and effects of difficulties in infertility provider-patient communication and relationships in the US and elsewhere, but strikingly, have not been examined in prior studies. Hence, these questions were investigated as part of a larger qualitative interview study exploring providers’ and patients’ decisions, attitudes and experiences concerning several critical aspects of IVF and pre-implantation genetic diagnosis (PGD), including sex selection [14], maternal age cut-offs [15], numbers of embryos transferred [16], reductions of multi-fetal pregnancies [17], diseases warranting PGD [18], insurance coverage [13], use of egg donor agencies [19], unconventional combinations and quality of prospective parents [20, 21], referrals for treatment [22], doctor-shopping [23], and religious issues that arise [24].

The present paper thus examines critical data that have not heretofore been probed or published, generated from questions regarding difficulties in clinician-patient relationships and communications – e.g., whether these perceived difficulties affect patients’ experiences, and if so, how. Specifically, while patients may feel dissatisfied, questions arise concerning why these tensions and gaps persist; how providers themselves see and experience these strains; whether clinicians are aware of these perceived deficits, and attempt to respond, and if so, how; what factors are involved; and whether these tensions might be addressed, and if so, how.

## Methods

Briefly, as described elsewhere, 37 semi-structured interviews of around one hour each were conducted with physicians and other providers involved with ART, and with patients [13–24].

Qualitative methods were used because they can optimally elicit the full range and types of views, relationships and practices involved, and can inform subsequent quantitative research. Qualitative methods have successfully elucidated key aspects of patient views and decisions regarding other aspects of IVF, such as those related to patients’ disclosures of use of donor oocytes [25].

Geertz [26] has suggested, from a theoretical standpoint, examining individuals’ lives not by imposing theoretical structures, but by attempting to comprehend the individuals’ own experiences and perspectives to obtain a “thick description.” The present study involved techniques of comparing data from different contexts for similarities and differences, to see if they suggest hypotheses. This technique generates new categories and questions, and checks them for reasonableness, and has been used in several other studies on key aspects of health behavior and doctor-patient relationships and communications in genetics and other areas [27–31]. During the ongoing interviewing process, the Principal Investigator (PI) continually considered how participants differed from or resembled each other, and the cultural, social, and medical factors and contexts that might contribute to variations.

### Participants

As seen on Table 1, 37 semi-structured telephone interviews of around 1 h each were conducted and analyzed. Both providers and patients were included to elucidate communication and relationships between these groups. Providers were recruited through national American Society of Reproductive Medicine meetings (e.g., PGD and mental health provider interest group meetings), word-of-mouth, and listservs. The PI approached individuals via these methods to see if they might be interested in participating in the study, and if so, the PI then sent them information about it. Most individuals whom the PI asked agreed to participate, and then did so. A mental health listserv was also used, received by approximately 60 members (not all of whom are active), of whom 15 responded, and the first 8 respondents then participated. Patients were recruited through providers, patient advocacy organizations, listservs, emails and word-of-mouth (e.g., via other patients). Interviewees were from across the United States. Interviews were conducted by phone and were transcribed. Since interviews were conducted by phone, not in person, an information sheet was sent to all participants who then provided verbal consent to participate. The Columbia University Department of Psychiatry Institutional Review Board approved the study and all the procedures involved.

**Table 1:** Characteristics of Sample

	Male	Female	Total
PHYSICIANS	14	3	17
Physicians who are also patients	0	1	1
Type of Practice			
University affiliated	5	1	6
Private Practice	9	2	11
OTHER ART PROVIDERS (e.g., mental health providers, nurses)	1	9	10
Other providers who are also patients	0	3	3
PATIENTS	1	9	10
TOTAL	16	21	37

### Instruments

The semi-structured interview questionnaire (Additional file 1) was drafted by drawing on prior literature, and explored patients’ and providers’ attitudes, decisions and experiences. The interview included both fixed questions, and follow-up questions to probe responses. The focus of the interview, about which patients were informed, was on experiences with infertility treatment, including interactions with providers.

### Data analysis

Transcriptions and initial analyses of data occurred during the period when interviews were conducted, and helped shape subsequent interviews. Once all interviews were completed, subsequent analyses were conducted in two phases, primarily by the PI and trained research assistants (RAs). In phase I, they independently examined a subset of interviews to gauge factors that affected participants’ experiences, identifying categories of “core” themes and issues that were then given codes. The RAs and PI read each interview, systematically coding sections of text to assign “core” codes or categories (e.g., instances of challenges in clinician-patient communication, and factors involved such as provider, patient and medical characteristics). While reading the interviews, a topic name (or code) was inserted beside each section of the interview to indicate the themes discussed. The RAs and PI then worked together to reconcile these independently developed coding schemes into a single scheme. A coding manual was then prepared, defining each code and examining areas of disagreement until consensus was reached. The coders discussed new themes that did not fit into the original coding framework, and modified the manual when deemed appropriate.

In the second phase, the coders independently content-analyzed the data to identify the main subcategories, and ranges of variation within each of the core codes. They reconciled the sub-themes each coder identified into a single set of “secondary” codes and an elaborated set of core codes. These subcodes assessed subcategories and other situational and social factors. Such subcategories included, for instance, specific types of medical, provider or patient characteristics that impeded provider-patient communication (e.g., large size of clinics, fears of lawsuits, or providers “hyping” their approaches).

Codes and sub-codes were then used in analyzing all interviews. Two coders analyzed each interview to ensure coding reliability. Multiple codes were used where necessary. Similarities and differences were assessed among participants, exploring categories that arose, variations within categories, and factors involved. Areas of disagreement were probed through further analysis until consensus was achieved. Earlier and later coded excerpts were regularly compared to check consistency and accuracy in ratings.

## Results

Interviewees were 27 ART providers: 17 physicians (MDs) and 10 other providers (OPs). These other providers consisted of seven mental health providers, two nurses and one patient advocacy organization worker. Ten patients (PTs) were also interviewed.

Providers discussed interactions with many patients and with colleagues. Patients frequently discussed interactions with multiple providers and other patients. Most patients had been undergoing infertility treatment for several years, and had tried several interventions unsuccessfully. Patients varied in their stage of treatment, seeking either first or second children. Among patients, seven worked (six full-time, and one part-time), two were graduate students, and one was unemployed. They ranged in age from 25 to 48 (mean: 36.1).

**Fig. 1:**
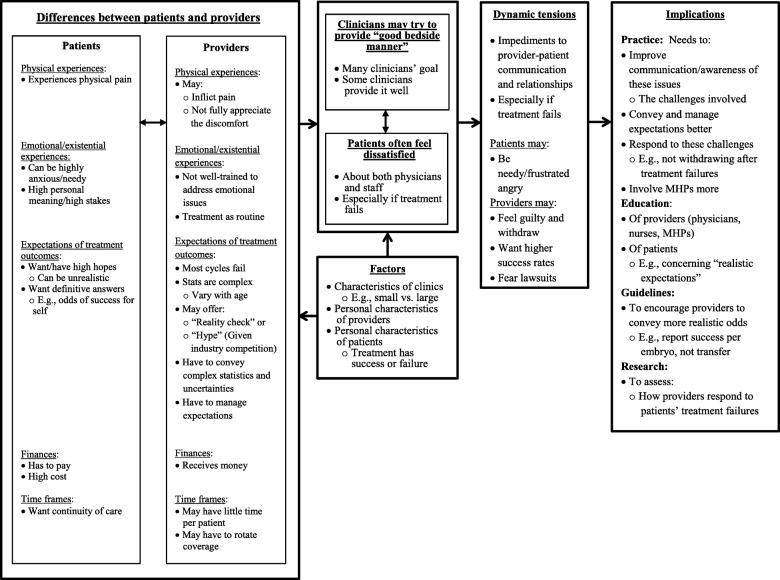
Issues Concerning Provider-Patient Communication and Relationships

As seen in Fig. 1, these data reveal several themes, suggesting how patients see clinicians’ interactions as ranging widely from good to bad, and how dynamic tensions between providers and patients result from several sets of differences between these two groups of individuals as well as characteristics of individual providers and patients. In brief, as described more fully below, contrasting experiences of treatment-related physical and emotional distress, outcomes, uncertainties, expectations, time frames and finances pose challenges that can hamper communication and relationships between these two groups.

### Differing perceptions of providers and patients concerning communication and relationships

#### Perceptions of providers as caring

Patients often have mixed, complex feelings about the quality of their communication and relationships with infertility providers. Many clinicians try their best to communicate and interact well with patients. Patients at times felt that their clinicians were well-informed, helpful and emotionally supportive. As one patient’s doctor said,

I just want to make sure you’re okay. How are you doing? What are you doing to take care of you? Are you going to therapy? Support group meetings? Talking to friends? [PT#8].

In working together to create new life, providers themselves can end up feeling closely connected to their patients. Many providers themselves find the work personally very rewarding, and become highly invested in the results.

It’s fun to watch those children grow. Many patients send me pictures every Christmas of their families as they grow. That’s been some of the joy – silent accolades, but they mean the most. I like to savor them. [MD#8].

Nurses, too, play critical roles, and may become very involved both professionally and emotionally in patients’ care. Nurses can come to feel closely connected to, and invested in, their patients.

I’ve lost sleep over patients I’ve grown strongly attached to. I really want them to be pregnant. During those 12 days after the transfer and before their pregnancy test, I’m sweating it out as much as they are. [OP#7].

Physicians, too, can become emotionally engaged with their patients. (“Patients bring us up or down.” [MD#9]) Many patients appreciate their doctors’ efforts, even when failure occurs. (“The most humbling letter we get is, ‘I didn’t get pregnant but thank you for taking care of me.’” [MD#9]).

#### Perceptions of providers as callous

Yet though many patients feel that their provider’s communication was at times sufficient, others felt disappointed. Doctors are dedicated, and generally attempt to help and prepare patients mentally for treatment difficulties, but may not always fully succeed. Given ongoing failures, patients frequently feel dissatisfied with both treatment difficulties and communication problems. Patients can feel a lack of empathy from clinics as a whole, including not only doctors, but nurses and other staff members as well. Even within clinics, however, variations can occur between providers.

I had an old-fashioned nurse… “Here are some needles. Go do this” …With the IUI, I felt she tried to give me false hope. With the miscarriage, I started bleeding. She said, “It happens. Just don’t worry about it. Come in for an appointment.” Another nurse, the first time I met with her, spoke about all the nuts and bolts, but said, “I just want you to know that I’m really sorry that this has happened to you...” I thought, “Thank God, somebody understands.” [PT#5].

The patient-centeredness of not only clinicians, but office staff – even receptionists – can be important. (“The bedside manner of the office and the practice helps – how the receptionist talks to you on the phone when you first call…Everything.” [MD#15]).

Patients may also see various providers and staff not individually, but as a whole, experiencing and referring to them as “they.” Patient dissatisfaction occurs particularly when treatment cycles have failed. Many such women may then feel that they are left alone to process the loss, and that providers are insensitive. Such patients may feel that they need more, but that they are then abandoned by their providers.

The first IVF did not work. They were very quick to push me into another one. They don’t give you time to digest it or do anything. I got pregnant, but had a miscarriage. They did not follow up with me…They just left me! [PT#6].

Other patients, disappointed and frustrated by the impersonality of care, may actually try to reach out and engage their physician, but may feel rebuffed. (“I had to push back and ask to talk to the doctor. I know they’re busy, but it’s important to have someone who knows and cares who you are.” [PT#7]).

When patients feel angry, they can end up lambasting doctors as well as staff. (“I’ve seen patients go off on the administrators at the front desk. A lot of patients treat staff very badly.” [OP#5]).

### Roles of differences between patients and providers

Several sets of differences between patients and providers – medical, physical, emotional; cognitive, logistical and financial – can contribute to perceptions of inadequate communication and relationships.

#### Differences in physical experiences of treatment

Patients – but not providers – experience and may complain about pain. Yet patients often see providers as ignoring these inherent difficulties and clinically-important complaints.

The doctors are basically surgeons, and a lot don’t have great bedside manner. The sixth week in the pregnancy, I had a sonogram, and then, two hours later had cramps. They seemed pretty bad, and I went back to the doctor, who made it seem like I was being histrionic. In fact, I ended up with an ovarian torsion and needed emergency surgery. I personally liked my doctor, but he handled that really poorly. [OP#9].

Patients may like their physicians, but nevertheless feel disappointed by aspects of care, underscoring the complexities involved in these relationships.

Indeed, providers who undergo fertility treatments themselves may come to appreciate far more fully than before the stresses and physical discomforts that patients face. Until becoming patients themselves, clinicians may thus dismiss patient complaints that these providers do not themselves experience and may thus find hard to grasp fully. One such physician-patient said:

I’m so much a better doctor than when I started. I’ve learned: instead of saying, “You’re just being a pain in the butt,” or, “You should stop talking,” or “I have to get out of here,” you need to just shut up and listen, and believe your patients. They’re not all lying to you! When I was having my first IVF attempt, lying there with my feet up in the stirrups as a patient, and they were sticking this giant needle through my vagina into my ovaries, and sucking out eggs, I would say every time they stuck the needle into my ovary “That hurts a lot.” The IVF specialist said, “Oh, the ovary doesn’t feel the pain, that’s your perineum.” He didn’t believe me!...When people said they had weird reactions to drugs, I didn’t believe them. But since then, I’ve had very strange reactions, too. No matter what the science says, every patient is individual. Give them the benefit of the doubt! [MD#15].

#### Differences in emotional/existential aspects of treatment

Infertility patients can experience complex and difficult emotions, yet feel that providers are insufficiently sensitive to these, reflecting in part lack of training and competing perspectives and promises. Patients felt that, given stresses patients encounter going through these procedures, clinicians could potentially be more sensitive in presenting and disclosing possible medical impediments to successful pregnancy.

Doctors are sincere, but maybe not properly trained on how emotionally fragile a woman is going through this. Doctors should never say, “Maybe there’s something wrong with your eggs.” It could be true, but patients don’t always need to hear the worst case scenario. I may not be able to handle it right now. Sometimes doctors are giving us too much credit: “here are the options.” [PT#7].

Patients may see themselves as posing challenges for providers, but tended to feel that providers could nonetheless try to be more aware of these difficulties.

The nurses are impatient with women in our situation, because we are high-maintenance, often calling a lot: “This is happening. Is this normal?” Pregnant patients are neurotic: “I have this. I feel a cramp here.” Nurses need some education about why it’s important to be sensitive, and have understanding and compassion for women going through this. [PT#7].

These women may be “fragile” in part because these treatments involve high stakes – the life of a possible future child.

Relatedly, having a child can provide important and unique personal meaning and purpose to patients, but can be largely “routine” and become “routinized” to providers. (“Doctors do this for a living. But for me, it’s my life!” [PT#5]).

Providers observe this difference as well.

There is a certain kind of day-to-day grind to it, because of the gravity of what we do. I can certainly get stuck in the tasks that need to get done. I have three embryo transfers that represent incredibly stressful days for those three couples. But it’s, yeah [nonchalantly], I have three embryo transfers. [MD#9].

Patients may feel wary of the sterile, mechanical procedures and routinization that pay little attention to the intense emotional aspects of the experience, though recognizing the reasons for this routinization. Still, providers and patients perceive their experiences of these procedures from dramatically different perspectives.

At a lot of places, you feel you’re in a baby factory. A huge emotional component is lost. It’s understandable – it’s their job, they do this every day, it becomes routine for them. But for the patient, it’s anything but. [PT#5].

#### Differing expectations concerning outcomes and uncertainties of treatment

Given desires for a child, physical burdens and costs of treatment, patients seek hope, and providers must frame and manage expectations. Yet clinicians face quandaries regarding how to respond to the inherent uncertainties and complex emotions involved – how to frame ambiguities about possible outcomes - and may come across either as overly-optimistic (“hyping” their services) or giving “reality checks”. Providers need to convey adequately to patients the relatively low success rate of IVF (to avoid giving patients overly high expectations of a “take-home baby”), the possible psychiatric and other side effects of fertility medications, and the emotional difficulties of “losing a child” if a miscarriage occurs. Yet patients may not want to hear, and may have had difficulty accepting this information.

Patients may feel that doctors both are not entirely forthcoming and may not want to give, or prepare patients for, bad news.

I wonder what they teach OB/GYNs about what to do when it looks like a woman will miscarry. Each time, it felt like the writing was on the wall, but nobody told me that the yolk sacs on the ultrasound weren’t the right size, or that the heart rate at seven weeks wasn’t what it should have been. We’re so numb; we hear part of that, but we weren’t told, “Brace yourselves. This is probably not going to go well.” Aren’t they supposed to tell you that they think you’re going to miscarry? Is that medically irresponsible to send a patient out waiting for a miscarriage? It seems like unless the heartbeat has stopped, they don’t completely tell you. [PT#10].

Complex statistics, in particular, can be hard to grasp, convey and apply for any one patient. Physicians may thus vary in how they communicate these odds to patients – what verbal descriptors and adjectives they employ.

A lot of what I try to do is manage expectations. A lot of providers use adjectives: high, low, moderate. That’s fine, but you’re not always on the same page. So using the absolute numbers is much more useful for patients. Some IVF providers say: “This procedure is very likely to be successful” – in their own universe, compared to 47-year-old women who have had ten miscarriages. But that may translate into only a 25% rate per cycle. It is likely to be successful for a woman with no infertility issues. [MD#6].

Patients generally seek not merely statistics, but ways of interpreting and making sense of these numbers. Patients often have trouble figuring out how to apply to themselves the varied statistics on averages that physicians provide.

Doctors have to use statistics, but sometimes I just want an honest opinion. Sometimes the doctors just give you the statistics. I finally had to say, “What would *you* do?” He told me, “If my daughter was going through this, *this* is what I would tell her to do.” It made me feel*,* “Okay, this is what I’m going to do”. [PT#9].

Patients may feel that statistics alone are not “honest” (i.e., straightforward), wanting instead a clear or definitive answer of how to proceed.

Providers face tensions of exactly how helpful to be, and in what ways, since many patients may wish for more direct assistance than providers may feel comfortable giving.

I just try to give patients realistic expectations, so they’re not disappointed. I gave a “reality check” to a 43-year-old woman who hasn’t yet found Mr. Right, and was trying to decide whether to use a sperm donor: “You’re 43. Even though you’ve never tried to get pregnant before, these are your odds: just being scientific about this, with one cycle of IVF and PGD, without any reproductive or medical issues, there’s only a 25% success rate. If you’re 43, those odds go down by an order of magnitude.” [MD#6].

Providers can face a hard balance to avoid being either too definite or not definitive enough. “Reality checks” may be appropriate, but be seen by patients as harsh, though certain patients may have unrealistic hopes.

The fact that considerable uncertainties exist concerning the outcomes of ART can also be hard for clinicians to convey, and for patients to grasp. Some patients felt that doctors seemed too definitive in decisions and predictions, and at times later ended up being wrong. As one patient said,

There’s a lack of complete knowledge here, which is very frustrating. There’s some science to it, but it’s not all science. It’s a little bit of hocus pocus. They wanted to trigger the cycle that led to me being pregnant with my daughter. I pushed back: “I think I should go another day. Maybe the eggs weren’t mature enough.” The doctor called and said, “If you want to go another day, go another day.” I said, “Should I? You’re the doctor.” He said, “If you don’t go another day, and it doesn’t work, you’re going to think that’s why. So I think you should just go another day.” He basically said: “This is an art *and* a science. It’s not just one of the other.” [PT#7].

Uncertainties can emerge, too because many patients have varying degrees of sub-infertility, rather than complete infertility per se, and can potentially become pregnant on their own but face diagnostically unclear medical impediments. Many patients may feel, however, that providers are insufficiently sensitive about these ambiguities.

The worst part is there’s some insensitivity in the field. Nurses, embryologists, or the REIs, will say, “Maybe there’s something wrong with your eggs or sperm.” They did a sperm test on my husband and said, “Something is abnormal here. You need to redo the test.” My husband hates doing sperm tests. So I said, “Just wait to the last minute on that.” The next month, I got pregnant on our own! [PT#7].

#### Differing time frames

Physicians confront many competing stresses, and may not have enough time with patients.

It would help if doctors gave a little bit more time to their patients, rather than just coming in, doing a sonogram, and going out. That’s hard, because these doctors have to see a lot of patients. But these patients need some emotional understanding. [OP#4].

Doctors in a clinic may also rotate being on-call, which can hamper continuity of care and disturb patients, but be inevitable. Still, patients may feel frustrated, though nevertheless understanding these limitations.

I went to an office with five fertility doctors. The doctor I was referred to, whom I wanted to see, wasn’t always my doctor. So I didn’t feel any one person was in charge of my treatment. He came up with the plan, but then the doctor on call that day decided the next step. That was very difficult. [PT #5].

The fact that patients must often pay all or much of the costs of treatment out-of-pocket exacerbates these tensions as well.

### Factors involved in characteristics of individual clinics, providers and patients

#### Institutional and professional characteristics of individual providers

The structure and logistics of clinics, care and professional roles can also create obstacles and pressures. The patient above, who complained about five doctors rotating in a clinic, added,

At a more private office, that wouldn’t have been the case. But this office is supposedly one of the best in the state, and is five miles from my house. So it was more realistic for me, but difficult. Still, I was very pleased with them. They were compassionate and skilled. One doctor, in particular, would sit for two hours if you needed, it, to go over every question you had. [PT#5].

Despite the frustrations she encountered, she ended up giving birth to twins, and looked back favorably on the experience overall. Depending in part on their own personal experiences, patients may be able to understand and appreciate the challenges that providers confront.

#### Personal characteristics of individual providers

Patients often see providers as varying widely in specific behaviors and characteristics regarding interactions, such as not only communicating effectively at the present time, but remembering details of such past interactions.

I don’t like when a doctor sits down and looks at my chart, trying to figure out who I am. Read my chart before you come into the room! I have had doctors who are really good at this: they have hundreds of patients, and probably don’t remember, but they seem to. That’s comforting. [PT#7].

Providers’ specific individual characteristics and experiences can shape their responses to these challenges. A variety of providers’ personal characteristics can be involved, but are not always readily predicted by simple or obvious objective categories such as gender.

It’s personality-dependent. Some men very closely watched what their wives have gone through, and can be compassionate and non-judgmental. But in general, I think women are a little bit more patient and less judgmental. Every woman has also had a period, and knows what that feels like. Yet *that* sometimes makes them more judgmental, because they tolerate their’s just fine, and just don’t believe other women who are in agonizing pain. It’s more personality than anything else. [MD#15].

Clinics and practices themselves also range in size and institutional cultures in ways that affect perceptions of care and potential tensions between providers and patients . Clinics vary in how they are organized, and structure interactions with patients.

At some facilities, you feel like cattle. You’re just being herded through. That’s what most facilities do. You go in for your blood work and sonogram and leave. The doctor reviews it after you’re gone. And they call you in the afternoon. You never have any doctor-patient contact until the transfer and the retrieval. [PT#7].

Several interviewees suggested that larger, multi-doctor practices may seem less caring than smaller ones. As one provider-patient who works in a small practice said, “In a very small clinic, we give people individualized care. I’ve had patients who were initially in a larger clinic, and felt like a number.” [OP#10].

Many patients concur, but may not always be wholly accurate and objective.

Multi-doctor practices are not there for the patient. Too many hands are in the pot. Too many people saying, “I see it going this way.” They don’t stick to the plan. With one-doctor offices, the doctor, even though you don’t get to see him all the time, makes himself available for you, and is more there for the patient: “It’s breaking my heart to see what you’ve gone through, and what you’ve spent when you should have just come to me from the beginning.” [PT#6].

Yet the doctor at this last clinic, though expressing empathy, may also be somewhat biased in suggesting that his treatment will be better than that of the patient’s prior physicians.

#### Characteristics of individual patients

As suggested above, medical, social, psychological, and financial characteristics of individual patients can also affect these tensions and experiences. Patients can vary in the type and strength of their emotional reactions, and needs for psychosocial support, related in part to the amount of treatment failure they have had, and their age and responses, which will affect how much opportunity they have to undergo additional IVF.

### Dynamic tensions

These differences can fuel dynamic tensions that become exacerbated when, for instance, treatments fail. When interventions do not succeed, patients may feel disappointed and angry and blame doctors, who themselves may feel frustrated, helpless and/or guilty, and thus withdraw or distance themselves. Doctors may have trouble discussing failures with patients, creating a vicious cycle. Many patients may understand and accept these failures (“A great reward is those people who in the end say, ‘We know we tried our best’” [MD#8]); but others have paid relatively large amounts of money, undergone physical burdens, and had high hopes dashed, and feel disappointed, shame, and anger. When strains, difficulties or failure occur in the treatment, patients can easily feel stressed and frustrated.

Women feel like pariahs if the cycle hasn’t worked, or they have an early miscarriage. Nobody really talks to them. Nurses are beginning to do that more. But doctors should reach out to the patient. It goes a long way to hear from the doctor – that the doctor is very busy, but actually cares. These women feel like failures, and that the doctor isn’t going to be very interested in them – because they failed, and haven’t contributed positively to their doctor’s success rate. [OP#4].

Clinicians may not respond well to these failures, and may distance themselves, worsening tensions.

The doctors may not want to reach out and then be blamed. Doctors can feel guilty that they failed, too. After pregnancy losses, a lot of the doctors feel very sad and guilty, even if there was no negligence or malpractice. And we’re a litigious society. A lot of patients can’t accept that bad things just happen. They blame the doctors. Sometimes it is the doctor’s fault, but not always. [OP#4].

Fears of lawsuits can further impede communication. (“Physicians may fear potential lawsuits. But I think if they show a humane approach, they’re less likely to be sued.” [OP#4]).

Poor communication between doctors and patients may result from mutual reticence.

The doctor may be reluctant to reach out to patients, too. Some patients can be very angry that it didn’t work, despite the doctor having very clearly told them their likelihood of success. [OP #4].

Alternatively, when confronting such treatment failures, many providers try to remain communicative, which can help maintain or improve PCC.

Families get frustrated when they don’t get answers, but doctors try their best. I’m not asking doctors to be friends, but to be available. Many call back, give the patient as much information as they can, and will do their best to help the family, and want to know updates: Did the PGD work? Was it successful? And they’re so happy when the patient has a healthy baby. [OP #7].

Several patients and providers suggested, in particular, that explanations of why communication is at times difficult can potentially help.

One receptionist was really stressed out, having an emergency situation going on, so she cut me off short. But as soon as she said, “We have an emergency. I need to call you back,” I totally understood. I know how emergencies go. But the other receptionists have all been good. [MD#15].

## Discussion

These data, the first to examine why IVF clinicians have difficulty communicating and interacting with patients, suggest that five sets of differences between providers and patients, and several characteristics of individual clinicians and patients can create dynamic tensions that impede communication and relationships. Providers and patients frequently respond differently to the medical issues involved, including high levels of uncertainty and complex statistics. Success rates are increasing, but still generally below 50%, particularly for older women [32]. Consequently, patients often have overly high expectations of success and definitive answers. Providers may either “hype” their success to attract patients, or offer reality checks – yet both of these approaches can generate tensions with patients. Patient can feel high levels of emotional distress, partly since they seek only one outcome (a “take home baby”), and view partial success (e.g., a pregnancy that ends in a miscarriage) as failure, while providers are very aware that most IVF cycles fail.

Patient complaints about provider interactions may not all be wholly accurate, but reflect these individuals’ feelings, and are thus important to note. Whether and to what degree these physicians are in fact having difficulties communicating and interacting with patients is unclear, but the fact that patients perceive such problems is critical, since these perceptions can affect whether and how patients pursue treatment.

While prior research has shown that patients often feel disappointed by communication and relationships with their providers [2–7], the present data suggest that several different specific barriers and factors exist, involving dynamic processes. While the past literature has suggested that clinics should address their organization, and have more frequent appointments and better quality of information for patients [2], the present data suggest several additional specific obstacles, details and aspects of potential improvements that have not been examined and should be addressed at several levels. Statistical and emotional uncertainties and complexities inherent in infertility treatment, and characteristics of particular providers, clinics, and patients can impede doctor-patient communication and relationships. The fact that several physicians described how they understood patients’ needs and perceptions only after becoming infertility patients themselves highlights needs to explore and articulate more fully these patient perspectives – the components, effects and importance of these viewpoints. Complex dynamic tensions and processes are involved. In general, physicians have been found to have difficulty coping with treatment failures. For instance, obstetricians have been found to feel guilty when patients have a stillbirth [33]; and physicians frequently feel guilty about medical errors, which can make these mistakes difficult to disclose to patients [34]. Yet physician non-communication can antagonize patients more than these doctors realize, aggravating patient frustration and ultimately harming the profession in the long run. Though pain is inherently hard to communicate to others [35, 36], physicians can become more aware of this difficulty itself. Patients may also not fully appreciate the pressures and stressors that providers themselves confront. Hence, enhanced patient appreciation of these clinician challenges could also potentially improve provider-patient interactions.

The current data suggest how the contexts of fertility care can also strongly affect several factors involved – e.g., the fact that in fertility treatment, patients seek and find acceptable only one acceptable outcome (a “take home baby”) rather than partial success (e.g., partial symptomatic relief of a chronic disease). Many patients pay all or significant proportions of treatment costs themselves, which may lead them to seek “the best” possible care, heightening desires for “good bedside manner.” In a competitive, entrepreneurial market, physicians may also overly “hype” their success. Issues of sexual behavior, reproduction and infertility are also extremely sensitive, and traditionally taboo to discuss [37], heightening needs for sensitivity. These data thus highlight the impact of contrasts in patients’ and providers’ particular roles in these medical, emotional, temporal, financial and institutional contexts.

Patients who end up with a child may ultimately look back at these experiences more favorably, but most cycles do not end up producing a baby [23]. Patients commonly undergo treatment over several years, filled with disappointments and struggles with costs and intervention failures, and perceive deficits in doctor-patient communication. Even patients who ultimately succeed and look back more favorably on the experience as a whole may perceive significant gaps in provider-patient relationships that hamper care. Several patients had had one child using ARTs and were now trying to have a second child.

These data have critical implications for future practice, research, guidelines and education. For practice, providers can address in several ways patients’ perceptions of gaps in communication and relationships. Particular aspects of clinics, including interactions with not only physicians but other providers as well, can shape the structure and organization of clinics, and can be improved. These data highlight how interactions, including seemingly small casual comments, can shape patients’ perceptions that their care is lacking. Nuanced approaches are needed to grasp how each group sees and responds to the other.

These data suggest, too, that physicians can involve mental health providers and non-physician staff more to benefit patients confronting treatment failures and other stresses, though these staff, too, need to be as aware and sensitive as possible to these issues. Many patients appear willing to understand these impediments, but providers may communicate about these obstacles and reasons poorly or not at all, fostering frustration. Clinicians may thus need to communicate better about these impediments, sensitively manage expectations about care, and address these processes and professional and personal obstacles at multiple levels. More attention to these issues and education by providers about these inherent uncertainties and realistic odds of treatment success are critical. Clinicians should realize that patients may have overly optimistic hopes, as well as difficulties hearing and grasping that the odds of success are overall less than 50% per cycle, and that uncertainties can remain.

These data suggest several questions for future research concerning how providers respond to ongoing treatment failures, how often clinicians withdraw, in what ways, and with what effect, what percentage of patients have unrealistic expectations, how, and to what degree. Future research can also investigate more fully the experiences of clinicians who become infertility patients themselves, to assess more thoroughly what specifically they now understand that they previously did not, and what barriers stymied this earlier appreciation.

These data also have key implications for education, highlighting needs to train physicians, other providers, patients, and the public to address more fully the emotional complexities with which these patients grapple, and needs to assist patients in developing realistic expectations. Though providers may fear being sued by patients for infertility treatment failure, patients in the US and elsewhere have sued infertility providers for various other reasons (e.g., accidental use of another patient’s gametes, loss or destruction of embryos, and birth of an “extra” child), but do not appear to have sued for failure to become pregnant [38–43]. Increased awareness of these facts can potentially help providers.

For policy, professional organizations can develop guidelines that encourage providers to avoid hype, and help patients develop realistic expectations. SART and CDC could also alter their requirements of what statistics clinics report, in order to aid patients more, providing more user-friendly breakdowns of data to assist patients in obtaining realistic rather than “hyped” understandings of the odds of success – e.g., by reporting success rates per embryo transferred, rather than only per IVF cycle (which can be higher if two or more embryos are transferred).

These data have several potential imitations. The sample size is adequate for qualitative research to reveal the themes and issues that emerge, but not for statistical analyses of how different groups (e.g. physicians vs. patients) may vary. Patients complain here about both private and hospital-based clinics, yet generally do not have sufficient experiences with both to generalize validly between them. Future studies can, however, investigate these issues with larger samples.

## Conclusions

These data, the first to examine why IVF clinicians have difficulty communicating and interacting with patients, highlight several key factors involved, and have critical implications for future practice, research, guidelines and education, highlighting needs to explore and address more fully these patient perspectives and dynamic processes.

## Additional file

Additional file 1: Semi-Structured Interview Questionnaire (Sample Questions). (DOCX 15 kb)

## Abbreviations

ART: Assisted reproductive technology
CDC: Center for Disease Control and Prevention
IUI: Intrauterine insemination
IVF: In-vitro fertilization
MD: Medical doctor
OB/GYN: Obstetrician/gynecologist
OP: Other provider
PCC: Patient-centered care
PGD: Pre-implantation genetic diagnosis
PT: Patient
REI: Reproductive endocrinology and infertility specialist
SART: Society for Assisted Reproductive Technology
