# Supplementary material for: Impediments to communication and relationships between infertility care providers and patients
Source: BMC Womens Health. 2018 Jun 5;18:84. doi: 10.1186/s12905-018-0572-6 (PMC5989459; doi:10.1186/s12905-018-0572-6)
Supplement: Supplementary file 1 — Semi-Structured Interview Questionnaire (Sample Questions). (DOCX 15 kb) [file 12905_2018_572_MOESM1_ESM.docx]

**Semi-Structured Interview Questionnaire (Sample Questions)**

Sample questions for *Providers* included:

- What challenges have you faced in your work as an ART provider?
  - How do you address these challenges?
- Have you faced challenges in communicating with patients? If so, when and how? What did you do?
- Have you faced challenges or obstacles in optimally caring for patients? If so, when, what and how? What did you do?
- How do you view these issues?
- What additional thoughts do you have about these issues?

Sample questions for *Patient*s included:

- What challenges have you faced as an infertility patient?
  - How do you address these challenges?
- Have you faced challenges in communicating with providers? If so, when and how? What did you do?
- Have you faced challenges or obstacles in relationships with providers? If so, when, what and how? What did you do?
- How do you view these issues?
- What additional thoughts do you have about these issues?
